# Pharmacokinetic Investigation of Commercially Available Edible Marijuana Products in Humans: Potential Influence of Body Composition and Influence on Glucose Control

**DOI:** 10.3390/ph14080817

**Published:** 2021-08-20

**Authors:** Taylor Russell Ewell, Kieran Shay Struebin Abbotts, Natasha N. Bondareva Williams, Hannah Michelle Butterklee, Matthew Charles Bomar, Kole Jerel Harms, Jordan Douglas Rebik, Sarah Margaret Mast, Natalie Akagi, Gregory P. Dooley, Christopher Bell

**Affiliations:** 1 Department of Health and Exercise Science, Colorado State University, Fort Collins, CO 80523-1582, USA; Taylor.Ewell@colostate.edu (T.R.E.); Kieran.Abbotts@colostate.edu (K.S.S.A.); Natasha.Williams@colostate.edu (N.N.B.W.); hmbutter@rams.colostate.edu (H.M.B.); Matt.Bomar@rams.colostate.edu (M.C.B.); kharms@rams.colostate.edu (K.J.H.); jrebik@rams.colostate.edu (J.D.R.); Sarah.Mast@colostate.edu (S.M.M.); 2 Department of Environmental and Radiological Health Sciences, Colorado State University, Fort Collins, CO 80523-1680, USA; natalie.akagi@gmail.com (N.A.); Gregory.Dooley@colostate.edu (G.P.D.)

**Keywords:** cannabis, cannabinoid, diabetes, insulin, delta-9-tetrahydrocannabinol

## Abstract

The purpose of the study was to describe and compare the pharmacokinetics of five commercial edible marijuana products, determine the influence of body composition on pharmacokinetics, and, in light of epidemiology suggesting marijuana may offer diabetes protection, explore the influence of edible marijuana on glucose tolerance. Seven regular users of marijuana self-administered five edible products in a randomized crossover design; each product contained 10 mg of delta-9-tetrahydrocannabinol (THC). Thirty minutes following marijuana ingestion, participants imbibed a 75 g glucose beverage. Time-to-peak plasma THC concentration ranged between 35 and 90 min; maximal plasma THC concentration (C_max_) ranged between 3.2 and 5.5 ng/mL. Differences between products in plasma THC concentration during the first 20–30 min were detected (*p* = 0.019). Relations were identified between body composition and pharmacokinetic parameters for some products; however, none of these body composition characteristics were consistently related to pharmacokinetics across all five of the products. Edible marijuana had no effect on oral glucose tolerance compared with a marijuana-free control (Matsuda Index; *p* > 0.395). Commercially available edible marijuana products evoke different plasma THC concentrations shortly after ingestion, but do not appear to influence acute glucose regulation. These data may allow recreational marijuana users to make informed decisions pertaining to rates of edible marijuana ingestion and avoid overdose.

## 1. Introduction

Legal access to marijuana, derived from the plant *Cannabis sativa* L., is increasing. To illustrate, within the USA, at the time of manuscript submission, marijuana is approved for recreational and medicinal use in 18 and 36 of the 50 states, respectively. Internationally, recreational use of marijuana is permitted in Uruguay, Canada, Georgia, and South Africa, and marijuana possession has been decriminalized in many countries, including most throughout South America. Accompanying increased access to marijuana is the number of options for mode of consumption. While inhalation of combusted marijuana remains common [1,2], the consumption of marijuana via commercially available edible (oral) products is becoming more prevalent. This increase in edible consumption is not without implication. The incidence of edible marijuana overdose reported to United States poison centers is also increasing (e.g., ~5-fold increase between 2013 and 2015), the symptoms of which range in severity from mild anxiety and lethargy to respiratory depression [3]. The increase in overdose prevalence has been attributed to two factors: accidental/unintentional ingestion (i.e., the patient was unaware they were ingesting marijuana) most common in children [4,5], and repeated premature dosing because of the absence of an immediate sensation of altered psychoactive state [6,7]. That is, compared with inhalation, the time to perceived effects of the edible marijuana are appreciably delayed [8]. Unfortunately, detailed studies describing the variability in the pharmacokinetics of the psychoactive component (∆-9-tetrahydrocannabinol (THC)) and its metabolites (11-hydroxytetrahydrocannabinol (THC-OH) and 11-nor-9-carboxytetrahydrocannabinol (THC-COOH)) from commercially available edible marijuana products are lacking.

Studies that have quantified circulating THC concentration following marijuana ingestion have yielded highly variable results [8,9,10]. Several explanations have been proposed to explain this variability, one of which includes differences in the composition of the edible product. For example, while additional ingredients and/or macronutrients within the product, such as the fat and/or protein in marijuana brownies, may enhance the flavor, they may also influence the rate of THC absorption from the gut. Additionally, the body composition of the edible marijuana consumer may have an influence on pharmacokinetics. Case in point, cannabidiol (CBD) is a non-psychoactive component of *Cannabis sativa* L.; the time to maximal circulating CBD concentration (T_max_) following ingestion of a commercial oral CBD preparation is related to fat free mass (R^2^ = 0.365) [11]. Further, adiposity may also influence the pharmacokinetics of THC absorption on account of the lipophilic properties of THC [12].

Aside from recreational use, marijuana and other *Cannabis sativa* L. products have been purported to have multiple medicinal benefits [13,14]. These include pain management [15], the treatment of Inflammatory Bowel Disease [16] and epilepsy [17], decreased nausea associated with some cancer medications [18], and potential protection from diabetes [19,20,21,22,23]. The latter claim is based on large epidemiological studies reporting on decreased incidence of diabetes in habitual and former marijuana users. With the exception of three conflicting studies completed in the 1970s [24,25,26], very few carefully controlled and laboratory-based investigations exploring the direct influence of marijuana on glucose control have been completed. These older studies involved intravenous THC administration and inhalation of combusted marijuana. Compared with *Cannabis sativa* L. plants grown in the 1970s, present day genetically modified strains and modern cannabis products typically have greater THC potency [27]. Further, intravenous administration is not reflective of contemporary marijuana use [1,2]. Additional explanations for the relative paucity of controlled, laboratory-based studies include the current legal and ethical complications associated with the administration of marijuana to human research participants. However, several investigators have developed creative solutions for the study of marijuana use in humans while remaining compliant with local and federal laws [28]. These have typically involved field-based studies of self-administration of marijuana, or “naturalistic observation” studies.

The current study has several aims (1) to describe the pharmacokinetics of circulating THC, two of its metabolites (THC-OH and THC-COOH), and the metabolite to parent ratio, following ingestion of commercially available edible marijuana products. This information will provide the recreational marijuana consumer with guidelines as to the timing of marijuana absorption and thereby may prevent overdose. (2) To compare the pharmacokinetics of several different commercially available edible marijuana products. This will also provide the recreational consumer with potentially important information as to differences between the strength and the pharmacokinetics of different products, even when standardized for THC content. (3) To determine the influence of body composition on THC pharmacokinetics. (4) To explore the potential acute influence of edible marijuana on glucose regulation.

## 2. Results

### 2.1. Participants

**Figure 1:**
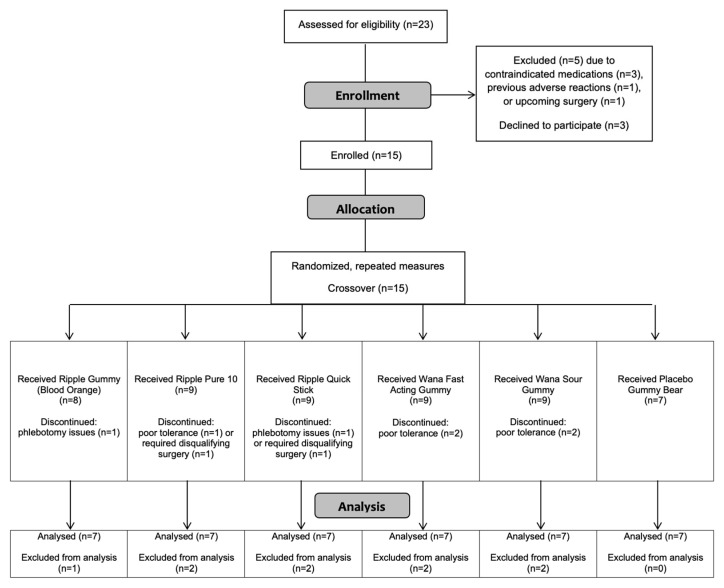
Consolidated Standards of Reporting Trials (CONSORT) flow diagram.

The progress of all participants throughout the trial (from screening and enrollment through to completion) is presented in Figure 1. A total of 15 participants were enrolled in the study, but only seven completed all procedures associated with the crossover design. Eight participants either withdrew or were excluded from the study for a variety of reasons that were not necessarily mutually exclusive. These included difficulties with phlebotomy (*n* = 2), an accident unrelated to the study requiring corrective surgery (*n* = 1), inability to refrain from marijuana use prior to study visits (*n* = 1), and voluntary withdrawal on account of poor tolerance of the research protocol (*n* = 4). Selected physiological characteristics of the remaining participants are presented in Table 1. Consistent with inclusion and exclusion criteria, the physiological characteristics were unremarkable.

### 2.2. Pharmacokinetics

**Figure 2:**
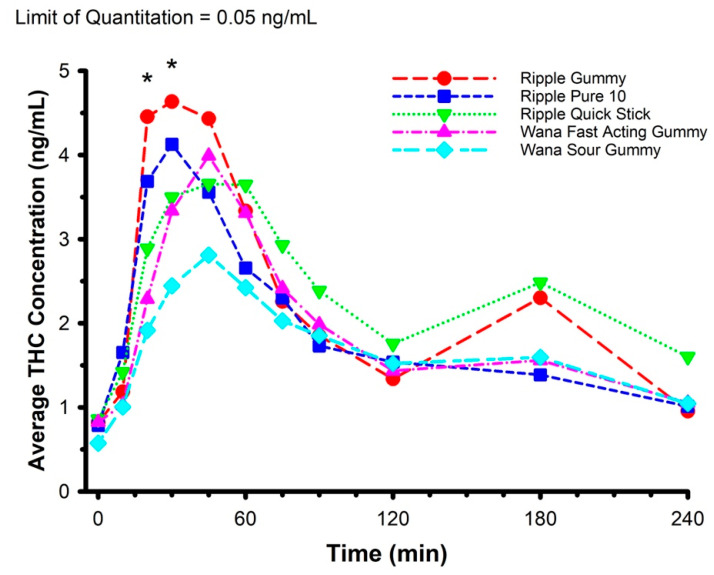
Mean circulating concentrations of THC following ingestion of commercially available edible marijuana (10 mg of THC). * Represents product × time interaction (*p* = 0.019). Post hoc analysis revealed circulating THC concentration was greater in Ripple Blood Orange Gummies vs. Wana Fast Acting Gummies (*p =* 0.003) and Wana Sour Gummies (*p* < 0.001) at 20 min, and greater in Ripple Blood Orange Gummies vs. Wana Sour Gummies (*p* = 0.002), and Ripple Pure 10 vs. Wana Sour Gummies (*p* = 0.038) at 30 min. Error bars have been omitted for clarity.

**Figure 3:**
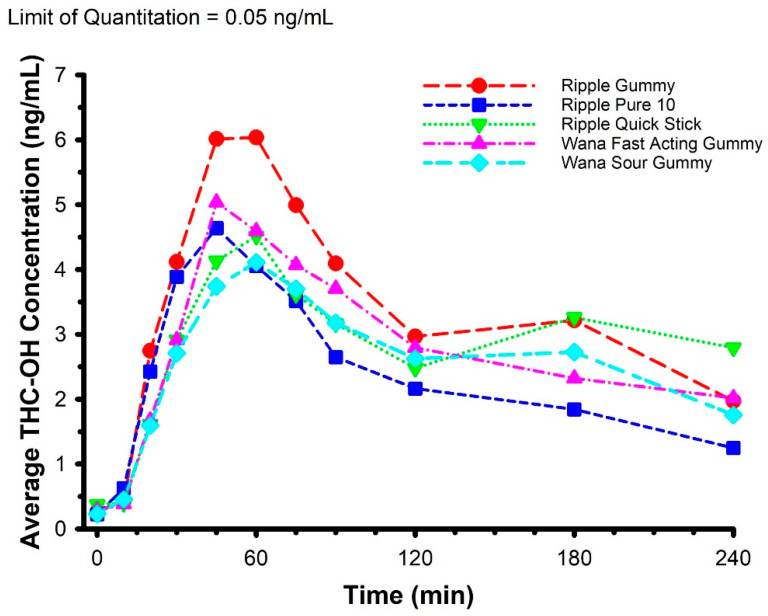
Mean circulating concentrations of THC-OH following ingestion of commercially available edible marijuana (10 mg of THC). There were no product × time interactions (*p* = 0.415). Error bars have been omitted for clarity.

**Figure 4:**
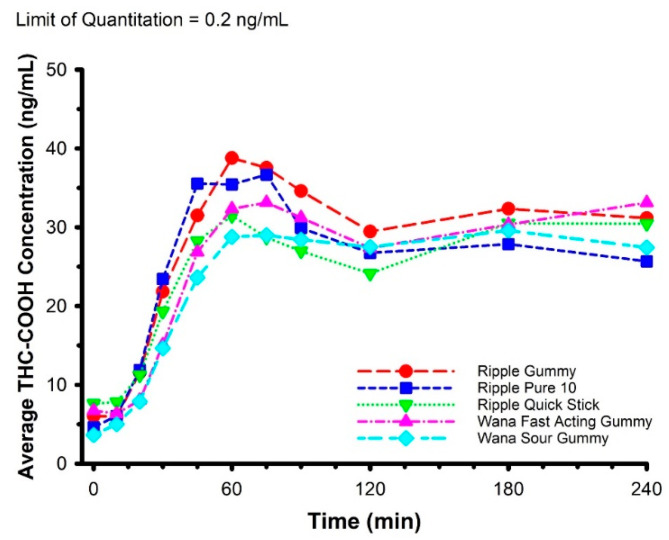
Mean circulating concentrations of THC-COOH following ingestion of commercially available edible marijuana (10 mg of THC). There were no product × time interactions (*p* = 0.485). Error bars have been omitted for clarity.

Features of each of the edible marijuana products are presented in Table 2. The mean circulating concentrations of THC, THC-OH, and THC-COOH are displayed in Figure 2, Figure 3 and Figure 4, respectively. For THC (Figure 2), there was a product × time interaction (*F* = 1.589, *p* = 0.019). Post hoc analysis revealed circulating THC concentration was greater in Ripple Blood Orange Gummies vs. Wana Fast Acting Gummies (*p* = 0.003) and Wana Sour Gummies (*p* < 0.001) at 20 min, and greater in Ripple Blood Orange Gummies vs. Wana Sour Gummies (*p* = 0.002), and Ripple Pure 10 vs. Wana Sour Gummies (*p* = 0.038) at 30 min. There were no product × time interactions for THC-OH (*F* = 1.038, *p* = 0.415) and THC-COOH (*F* = 0.995, *p* = 0.485).

Pharmacokinetic parameters for THC, THC-OH, and THC-COOH are displayed in Table 3, Table 4 and Table 5, respectively. None of the comparisons between edible products yielded appreciable differences for any of the pharmacokinetic parameters (all *p* > 0.06). Metabolite-to-parent ratios for C_max_ and AUC_0-240_ are presented in Table 6. There were no appreciable differences (all *p* > 0.13) for any of the comparisons.

### 2.3. Body Composition

Correlations between parameters of body composition and pharmacokinetics are presented in Table 7. While significant relations were identified between body composition and pharmacokinetic parameters for some products, none of these body composition characteristics were consistently related to pharmacokinetic parameters across all five of the products.

### 2.4. Oral Glucose Tolerance

**Figure 5:**
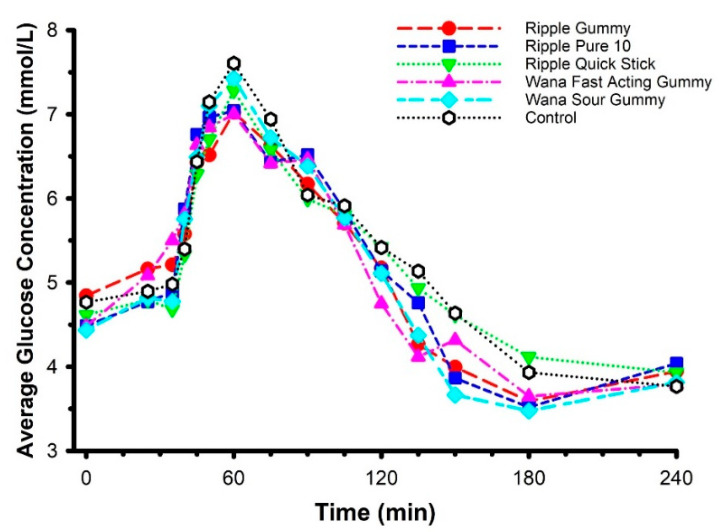
Mean circulating glucose concentrations following ingestion of commercially available edible marijuana (10 mg of THC) and one marijuana-free control product. Seventy-five grams of glucose was ingested at 30 min. Fasting glucose was not different across study sessions (*p* = 0.40). Circulating glucose was not different from fasting glucose 30 min after product ingestion (*p =* 0.88). Compared with placebo, none of the edible marijuana products influenced circulating glucose throughout each of the trials (product × time interaction *p* = 0.98). Error bars have been omitted for clarity.

Mean circulating glucose concentrations across the six conditions (including marijuana-free control) during an oral glucose tolerance test (OGTT) are displayed in Figure 5. Seventy-five grams of glucose was imbibed as a beverage 30 min after ingestion of marijuana (or control). Fasting glucose was not different across laboratory visits (*F* = 1.066, *p* = 0.40). Circulating glucose was not different from fasting glucose 30 min after product ingestion (*p =* 0.88), implying that the carbohydrate content of the edible products was too low to evoke an appreciable hyperglycemic response. Compared with marijuana-free control, none of the edible marijuana products influenced circulating glucose throughout each of the trials (product × time interaction *F* = 0.656, *p* = 0.98). Consistent with this observation, none of the edible marijuana products influenced other indices of the glucose response, including 2-h AUC (*F* = 0.142, *p* = 0.98; data not shown) and 3.5-h AUC (*F* = 0.290, *p* = 0.92; data not shown).

**Figure 6:**
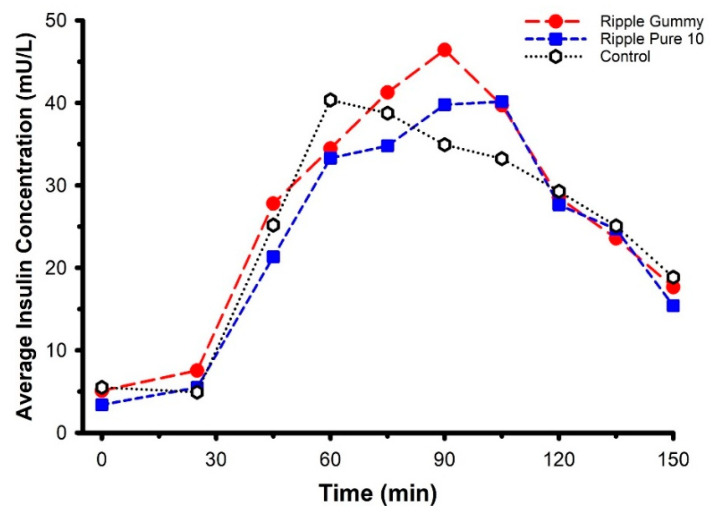
Mean circulating insulin concentrations following ingestion of two commercially available edible marijuana (10 mg of THC) and one marijuana-free control product. Seventy-five grams of glucose was ingested at 30-min. There were no differences in circulating insulin between products (product × time interaction *p* = 0.74). Error bars have been omitted for clarity.

An absence of difference in circulating glucose does not necessarily imply an absence of difference in glucose regulation. Accordingly, circulating insulin concentration was determined for two of the products—Ripple Blood Orange Gummies and Ripple Pure 10—and compared against the marijuana-free control (Figure 6). These two specific products were selected on account of the higher plasma THC concentrations evoked by their ingestion (Figure 2). Consistent with the glucose data, there were no differences in circulating insulin between products (product × time interaction *F* = 0.763, *p* = 0.74) and 2-h AUC (*F* = 0.372, *p* = 0.70) nor were there any differences in glucose/insulin derived indices of glucose regulation, including HOMA-IR (*F* = 1.366, *p* = 0.30; data not shown) and the Matsuda Index (*F* = 1.021, *p* = 0.40; data not shown).

## 3. Discussion

The primary findings of the current study were (1) THC T_max_ ranged between 35 and 90 min, and THC C_max_ ranged between 3.2 and 5.5 ng/mL. (2) Differences between products in plasma THC concentration during the first 20–30 min were detected. There were no differences between products in the metabolite-to-parent C_max_ and AUC ratios for THC-COOH and THC-OH. (3) Significant relations were identified between body composition and pharmacokinetic parameters for some products, however none of these body composition characteristics were consistently related to pharmacokinetic parameters across all five of the products. (4) Compared with a control condition, edible marijuana had no effect on any of the indices of glucose regulation, including Matsuda Index, and 2.0 h and 3.5 h AUC.

Commercially available edible marijuana products are becoming increasingly popular alternatives to inhaled combusted marijuana, in part due to the perceived lower cardiopulmonary risk associated with protecting lungs from exposure to smoke. However, as with marijuana prepared for burning/smoking, considerable pharmacokinetic variability exists between edible marijuana products, even when standardized for THC content. For example, in two recent studies of cannabis-infused brownies containing 50 mg of THC, THC C_max_ ranged between 2.5 ng/mL and 4 ng/mL, and T_max_ between 1 and 2 h [9,10]. In contrast, in the present study research participants self-administered commercially available edible marijuana containing only 10 mg of THC, and C_max_ ranged between 3.2 and 5.5 ng/mL, while T_max_ ranged between 35 and 90 min. Thus, several of the commercially available preparations containing only 20% of the THC content of the brownies evoked greater circulating concentrations of THC, and in a shorter period. To account for these discrepancies between studies, we have considered several potential explanations. In addition to cannabis, the brownies contained additional ingredients, including fats and sugars, that contributed to a greater caloric load compared with a single (10 mg THC) serving of any of the commercial edible marijuana studied in the current investigation; we conservatively estimate 100 vs. 0–30 kcal, respectively. It is plausible that the additional caloric load of the brownies interfered with the rate and amount of THC absorption from the gut [29]. Second, the brownies were typical/normal food supplemented with cannabis, whereas the commercial products under investigation were engineered to provide superior delivery of THC. This engineering includes the development of water-soluble THC and marijuana concentrate, and controlled addition of solubility agents, such as gum arabic and medium-chain triglycerides (MCTs), that have been shown to promote delivery and bioavailability of dietary supplements [30,31]. Third, in a separate brownie study [8], approximately 50 mg of THC incorporated within a brownie produced a THC C_max_ (measured in whole blood) of 4.7–34.8 ng/mL in frequent marijuana users (≥5 times per week) and 3.6–22.5 ng/mL in occasional marijuana users (≤3 times per week). In the same study, THC T_max_ was 1.5–3.5 h for both frequent and occasional users. These data imply that the degree of habitual marijuana use may promote THC C_max_ independent of T_max_, thus when comparing across studies, data must be interpreted with caution if habitual marijuana use is not reported. In the current study, a repeated measures crossover design was employed, thus comparison within the study should not be compromised by heterozygous habitual cannabis/marijuana use; this is a strength of our study.

Previously, it has been reported that parameters of oral CBD pharmacokinetics, including T_max_, were associated with anthropometric variables, including fat-free mass and body mass index [11]. A number of explanations were proposed, such as the positive relationship between fat-free mass and blood volume [32,33], representing a greater reservoir in which to dilute circulating metabolites; greater perfusion of metabolically active tissues, thereby leading to enhanced clearance of circulating metabolites; and the lipophilic nature of CBD. In the current study we extend these observations and arguments to consider a potential role of body composition specific to THC pharmacokinetics. To our knowledge, our data are the first to directly address this issue. While significant relations were identified between body composition and pharmacokinetic parameters for some of the edible marijuana products (Table 7), none of these body composition characteristics were consistently related to pharmacokinetic parameters across all five of the studied products. Similar to CBD, THC is thought to be lipophilic and consequently absorbed by adipose tissue. Two lines of evidence from frequent marijuana users support this claim: THC is quantifiable in fat biopsies taken 4 weeks after previous marijuana use [12] and circulating THC concentration is increased following 35 min of lipolysis-inducing moderate intensity stationary cycle ergometer exercise [34]. Similar exercise-mediated observations have been reported in exercising rats pre-treated with THC [35], but not reproduced in a smaller follow-up study of humans with low-to-recommended body mass index (BMI; range: 19–23 kg/m^2^) [36]. Our analysis revealed inconsistent correlations for absolute and/or % fat mass for T_max_, C_max_ and AUC_0-240_ for only some of the products. Although our study population was small, the range in adiposity and BMI was large, ranging from lean to obese. The heterozygous nature of the body composition data should have facilitated the identification of potentially significant correlations. While it appears likely that in vivo THC is lipophilic and absorbed by adipose tissue, adipose may not appreciably contribute to short-term edible marijuana pharmacokinetics.

The final aim of the current study was to explore the potential acute influence of edible marijuana on glucose regulation. The connection between marijuana use and the development of insulin resistance and diabetes is not clearly understood. On one hand, there exists an intuitive link: marijuana use is associated with increased appetite [37], leading to increased dietary intake, and in particular increased purchase of foods of high caloric but poor nutritional value (“junk food”) [38]. If this behavior is chronic then weight (fat) gain typically ensues, and the overweight and obese states are causally tied to insulin resistance and diabetes. However, on the other hand, most of the epidemiological research suggests that marijuana use either has no effect on diabetes risk, or in many cases lowers the risk of diabetes, even when taking into account other potential confounding variables such as body composition [19,20,21,22,23]. The collective concluding recommendation provided by the authors of these studies was the need for randomized controlled trials to provide definitive insight. To our knowledge, aside from some preliminary work completed in the 1970s [24,25,26], there have been no human intervention studies to support the epidemiology. The early preliminary work involved intravenous marijuana administration and inhalation of combusted marijuana, and resulted in either impaired or unaltered acute glucose regulation. Inhalation of combustible materials typically leads to temporarily decreased oxygen transport (i.e., relative hypoxia) and increased sympathoadrenal activity, two conditions known to unfavorably modify glucose control [39,40]. Further, since these early studies, the THC potency of marijuana products has increased [27] and the methods of marijuana consumption have become more diverse [1], thus our goal in the present study was to update the paradigm using commercially available edible marijuana with THC doses reflective of contemporary use. The gold standard measurement of glucose regulation and insulin sensitivity is the hyperinsulinemic euglycemic clamp technique, involving the intravenous co-administration of insulin and glucose. Unfortunately, on account of state laws and institutional regulations, marijuana is not permitted in our university clinical research facility, thus we compromised by studying glucose regulation using a modified OGTT in a field setting. None of the five commercially available edible marijuana products influenced the acute circulating glucose response to standardized glucose ingestion (*p* = 0.98). The variability in circulating glucose across conditions was akin to desirable studies of test–retest reliability [41]. The primary hormone involved in glucose regulation is insulin. Improvements in glucose control are not always necessarily reflected by lower circulating glucose concentrations [42]; improved glucose regulation may be reflected by lower insulin in the absence of modified glucose values. For this reason, we compared the OGTT insulin response in the control condition to the OGTT insulin response to two of the five edible marijuana products. There were no differences in circulating insulin between products, nor were there any differences in glucose/insulin derived indices of glucose regulation, including the Matsuda Index. Based on our data, and in light of the epidemiological studies [19,20,21,22,23], we suggest that, in adults free from diabetes, edible marijuana does not affect acute glucose regulation, but potentially may exert a favorable chronic influence via long-term physiological responses such as decreased adiposity [43], and improved gut [44] and/or liver health [45]. Noteworthy, recent data from a Mendelian randomization study suggest that chronic use of products derived from *Cannabis sativa* L. does not influence the prevalence of type 2 diabetes [46]. Clearly the potential connection between marijuana and glucose regulation remains unresolved.

There are several issues and potential study limitations that warrant further discussion. Marijuana and other *Cannabis sativa* L. products have been purported to have multiple medicinal benefits; the evidence, or lack thereof, to support some of these claims has recently been reviewed [13,14,47,48]. Collectively, these reviews highlight some of the inconsistencies between previous reports and conclude with recommendations for future research and the need for randomized controlled trials. In this regard, marijuana research is difficult. Among some of the problems unique to marijuana research are significant legal and regulatory compliance issues, difficulties associated with research participant compliance pertaining to short-term marijuana abstention, and ethical and safety concerns pertaining to the consumption of psychoactive substances. In addition, specific to marijuana research in the USA, there is currently a restricted number of federally approved cannabis growing facilities, thereby limiting the diversity of strains of plants with varied cannabinoid ratios and potencies available for study, and the requirement of a federal license and registration with the Drug Enforcement Administration for the dispensing of marijuana to research participants. With reference to the current investigation, marijuana is not permitted in our university clinical research facility and investigators are not permitted to handle marijuana. Study limitations resulting from these regulations included the need to transfer research location from a clinical facility to a field location, and compromise with the choice of procedures (e.g., the hyperinsulinemeic euglycemic clamp technique vs. the OGTT). With respect to the former, several of the phlebotomy problems that contributed to participant withdrawal may have been avoided, and the clinical furniture (e.g., adjustable hospital bed) may have promoted participant comfort during the 5 h protocols. As for assessment of glucose regulation, although not the gold standard protocol, the OGTT is generally considered an acceptable alternative to the clamp technique [49]. Additional study limitations include the number of participants who completed the whole study (7/15). While relatively small, the sample size was sufficiently large to allow for detection of differences in circulating THC concentration across marijuana products, and for the identification of significant associations between pharmacokinetic parameters and body composition variables. We did not detect an influence on glucose regulation between any of the marijuana products and the control products; while it is feasible that this could be attributed to a type 2 error, power analysis [50] suggested that based on our current data, and assuming a similar degree of variability, approximately 200 participants would have been required to detect an influence of marijuana on the Matsuda Index. Noteworthy, we studied young adults without diabetes. It is plausible that marijuana may have had a more pronounced effect on glucose regulation in adults with poor glucose control, including those with diabetes, or adults at risk for diabetes, such as older adults [51], and/or sedentary adults with body mass index values greater than 25 (overweight) or 30 kg/m^2^ (obese) [52]. In light of the difficulties associated with marijuana research, an alternative approach could be to persuade regular users of marijuana to abstain for a brief period, and quantify glucose regulation before and after abstention, and again after resumption of normal use. The hypothesis would be that glucose regulation was unfavorably influenced by marijuana abstention. Such an approach would circumvent the need to administer marijuana, but compliance with abstention, confirmation of compliance, and the substitution of marijuana for another behavior may introduce new challenges and/or confounding variables.

Limitations aside, we believe our data to be novel and of practical value. To our knowledge, our study represents the first to compare the pharmacokinetic properties of commercially available edible marijuana products and to evaluate the potential influence of body composition. Further, our data may be used by investigators to plan the timing of measurements and procedures in future studies exploring the physiological effects of edible marijuana.

In summary, in recreational users, knowledge of pharmacokinetics associated with different commercially available edible marijuana products may prevent accidental overdose due to premature repeat dosing. THC T_max_ and C_max_ ranged between 35 and 90 min, and between 3.2 and 5.5 ng/mL, respectively. Differences exist between commercial products with respect to plasma THC concentration during the first 20–30 min following ingestion. Body composition does not appear to be an acute determinant of edible marijuana pharmacokinetics. Edible marijuana does not appear to affect acute glucose regulation, but in light of epidemiological data, marijuana potentially may exert a favorable influence on long-term glucose regulation and diabetes risk.

## 4. Materials and Methods

The study was conducted according to the guidelines of the Declaration of Helsinki and approved by the Institutional Review Board of Colorado State University (Protocol #20-10278H, 8 October 2020). All participants provided written informed consent prior to commencement of the study. This study utilized a randomized, repeated measures crossover design.

### 4.1. Participants

Adult men and women aged 21 or over were invited to participate. Inclusion criteria included body mass greater than 50 kg, regular use of marijuana (≥four times in the previous month), willingness to abstain from all products derived from *Cannabis sativa* L. during the four days prior to each data collection, and previous use of a *Cannabis sativa* L. product containing ≥10 mg of THC without a significant adverse reaction. Exclusion criteria included pregnancy, breastfeeding, treatment for psychosis, bipolar disorder or schizophrenia, current or previous use of medication for treatment or prevention of diabetes, previous diagnoses of heart disease, peripheral vascular disease, high blood pressure, stroke, or heart murmur, and/or use of medication contraindicated for concurrent use with marijuana or known to influence glycemic control.

### 4.2. Protocol Overview

To remain compliant with institutional and state laws pertaining to marijuana, an observational approach was employed in a manner inspired by previous reports [28]. Following screening, on six mornings, each separated by a minimum of 1-week, participants self-administered one of five edible marijuana products or a marijuana-free control product in a randomized crossover design; each marijuana product contained 10 mg of THC. Thirty minutes following marijuana (or marijuana-free control) ingestion, participants imbibed a carbohydrate beverage containing 75 g of glucose. Venous blood was sampled repeatedly over 4-h and was analyzed for circulating concentrations of THC, THC-COOH, THC-OH, glucose, and insulin.

### 4.3. Procedures

Prior to study enrollment, potential participants completed a detailed electronic medical history questionnaire. Responses requiring additional query were addressed either in-person, via telephone, or video conference. Body size and composition were assessed at Colorado State University using dual energy X-ray absorptiometry (DEXA; Hologic, DiscoveryW, QDR Series, Bedford, MA, USA), and a physician’s digital scale and stadiometer.

The remaining data collection sessions were completed off-campus on six mornings, each separated by 1–2 weeks depending on participant schedules. The time of protocol initiation was kept constant for each participant. Every data collection was preceded by a 12-h fast, 24-h abstention from alcohol and exercise, and 96-h abstention from any products derived from *Cannabis sativa* L., including CBD and marijuana.

A venous catheter was introduced to an antecubital or dorsal hand vein and blood (~10 mL) was collected for analysis of baseline circulating concentrations of THC, THC-COOH, THC-OH, glucose, and insulin. Immediately following baseline blood collection, participants self-administered one of five edible marijuana products or a marijuana-free control product (described in detail in a subsequent section). Thirty minutes following marijuana (or marijuana-free control) ingestion, participants imbibed a beverage consisting of 75 g of glucose dissolved in 250 mL of water (i.e., an oral glucose tolerance test (OGTT)).

Relative to marijuana ingestion (Time 0), venous blood was sampled for subsequent analysis of circulating concentrations of THC, THC-COOH, and THC-OH at minutes 10, 20, 30, 45, 60, 75, 90, 120, 180, and 240. Blood was immediately transferred into chilled tubes coated with ethylenediaminetetraacetic acid (K3 EDTA) and placed on ice for up to 30 min before isolation of plasma via chilled (4 °C) centrifugation. One milliliter aliquots of plasma were then placed on ice while being transported to the research facility for storage at −80 °C prior to subsequent analysis.

Relative to marijuana ingestion (Time 0), the carbohydrate beverage was imbibed at minute 30. Venous blood was sampled for subsequent analysis of circulating concentrations of glucose at minutes 25 (i.e., post-marijuana but pre-glucose), 35, 40, 45, 50, 60, 75, 90, 105, 120, 135, 150, 180, and 240, and at minutes 25, 45, 75, 105, 135, and 150 for subsequent analysis of plasma insulin concentration. Blood intended for glucose analysis was transferred to chilled tubes containing sodium fluoride (potassium oxalate), and then immediately placed on ice for transport to the research facility where it was evaluated, in duplicate, without delay using an automated analyzer (YSI 2900 STAT Glucose Lactate Analyzer, YSI Inc., Yellow Springs, OH, USA). Blood intended for insulin analysis was processed in an identical manner to the blood used for THC, THC-COOH, and THC-OH analysis. Plasma insulin concentration was determined in triplicate via enzyme-linked immunosorbent assay ((ELISA) Crystal Chem, Inc., Elk Grove Village, IL, USA).

### 4.4. Commercially Available Edible Marijuana Products

Five commercially available edible marijuana products were selected for study. These products were made and sold at licensed stores throughout Colorado, USA. Participants were requested to purchase each of the identified products using their personal funds. Confirmation of purchase was verified by inspection of retail receipt. Features of each of the edible marijuana products are presented in Table 1. Each of the products contained 10 mg of THC, except for the marijuana-free control product. The marijuana-free control product was provided by the research team (i.e., purchase by participants was not required). All products were consumed within 30 s of self-administration. The order of self-administration was dictated by the research team based on a random generator.

### 4.5. Reagents and Supplies

THC, THC-COOH, THC-OH, THC-D3, THC-COOH-D3, and THC-OH-D9 were purchased from Cerilliant (Round Rock, TX, USA). A second set of THC, THC-OH, and THC-COOH was purchased from Lipomed (Cambridge, MA, USA) to be used for quality control samples. Water and acetonitrile (LC–MS-grade) were obtained from Millipore (Burlington, MA, USA). Dansyl chloride, sodium bicarbonate, sodium carbonate, acetic acid, and formic acid (LC–MS-grade) were obtained from Sigma-Aldrich (St. Louis, MO, USA). Captiva EMR-Lipid columns (1 mL, 40 mg) were purchased from Agilent Technologies (Santa Clara, CA, USA). Chromatography was performed with a Kinetex Phenyl Hexyl column (3.0 × 50 mm, 2.6 μm) purchased from Phenomenex Inc. (Torrance, CA, USA).

### 4.6. Calibrators, Quality Controls, and Internal Standard Preparation

Matrix matched calibrators and controls were prepared by the addition of appropriate volumes of methanolic stock standard mixes to 300 μL of cannabinoid free plasma. Working standard mixes containing 0.01, 0.1, or 1.0 μg/mL of THC, THC-OH, and THC-COOH were prepared from stock standards obtained from Cerilliant. They were used to produce calibrators for THC and THC-OH at 0.05, 0.1, 0.2, 0.5, 1, 5, 10, and 50 ng/mL and calibrators for THC-COOH at 0.2, 0.5, 1, 5, 10, and 50 ng/mL. Quality control samples were prepared at 0.7, 7 and 20 ng/mL for each analyte using working standard mixes of 0.01, 0.1 or 1.0 μg/mL of THC, THC-OH and THC-COOH. These working standards were prepared from stock standard obtained from Lipomed to verify the calibrators prepared from Cerilliant stock standards. Quality control samples were run after every 20 subject samples with an expected accuracy of +/−20%. The internal standard mix solution contained 30 ng/mL THC-D3, 100 ng/mL THC-OH-D3, and 300 ng/mL THC-COOH-D9 in methanol.

### 4.7. Cannabinoid Analysis by LC-MS/MS

Plasma samples and matrix-matched standards and quality controls were prepared for LC-MS/MS analysis by protein precipitation, lipid removal, and derivatization with dansyl chloride. Ten microliters of internal standard solution were added to 300 μL of plasma sample and mixed in a microcentrifuge tube. Nine-hundred microliters of acetonitrile containing 1% formic acid were added and vortexed for 30 s to precipitate proteins. Samples were centrifuged and supernatants transferred to Captiva EMR-Lipid columns for lipid removal. Using a positive pressure manifold, 3 psi of pressure was applied to the samples to elute through columns. Eluents were collected into a clean glass test tube and dried under nitrogen at 40 °C prior derivatization. Dried eluents were reconstituted in 100 μL of 1 mg/mL dansyl chloride in acetonitrile and transferred to autosampler vials fitted with 400 μL glass inserts. One-hundred microliters of a 0.1 M sodium carbonate bicarbonate buffer (pH 10) were added and the sample incubated at 55 °C for 20 min to derivatize the analytes. Samples were cooled to room temperature and neutralized with 10 μL of acetic acid prior to LC-MS/MS analysis.

Samples were analyzed with an Agilent 1290 UHPLC coupled to an Agilent 6460 triple quadruple mass spectrometer equipped with an Agilent Jet Stream electrospray ionization source (Agilent, Santa Clara, CA, USA). Cannabinoids were first chromagraphically separated on a Phenomenex Phenyl Hexyl column (3.0 × 50 mm, 2.6 μm) held at 40 °C. A sample volume of 10 μL was injected and a mixture of water with 0.1% formic acid (A) and acetonitrile with 0.1% formic acid (B) at a flow rate of 0.4 mL/min. The gradient elution used was 40% B for 0.5 min, increasing to 80% B at 2 min, increasing to 100% B at 4.5 min, and held at 100% B for 1.5 min. The ionization source conditions used were as follows: positive polarity, nebulizer 45 psi; gas flow of 10 L/min at 300 °C; sheath gas flow of 12 L/min at 390 °C; capillary voltage of 3500 V; nozzle voltage of 200 V. The ion transitions monitored are displayed in Table 8. Analytes were confirmed by retention time and the product ion ratio correlation between the sample peaks and corresponding standards (±20%). The data collection and processing were performed by using Agilent MassHunter Quantitative software (v.B.08.01). Quantitation was performed with linear regression using 8-point calibration curves from 0.05 ng/mL to 50 ng/mL for THC and THC-OH. A 6-point calibration curves from 0.2 ng/mL to 50 ng/mL was used for THC-COOH. Analytical staff were naïve as to the edible marijuana products (i.e., blind to specific products and conditions).

### 4.8. Pharmacokinetic and Oral Glucose Tolerance Test Analysis

Pharmacokinetic analysis of the circulating concentrations of THC, THC-OH, and THC-COOH for each of the products was completed using dedicated software (PhoenixWinNonlin v8.3, Certara, NJ, USA). Areas under the concentration curves were calculated using the linear trapezoidal method.

Glucose and insulin data were processed using established methods. These included calculation of Homeostatic Model Assessment for Insulin Resistance (HOMA-IR) [53] and Matsuda Index [54]. Areas under the concentration curves for 2-h (standard practice) and for 3.5 h (practice specific to the current study) were calculated using the trapezoidal method.

### 4.9. Statistical Analyses

All data, unless otherwise stated, are expressed as mean and standard deviation. Statistical calculations were performed using dedicated software (SigmaStat 3.0, Systat Software Inc., San Jose, CA, USA). Differences in circulating concentrations of THC, THC-OH and THC-COOH, glucose, and insulin over time and between products were examined using 2-way analysis of variance (ANOVA; product × time), with repeated measures (time). Differences in the pharmacokinetic properties between the edible marijuana products were examined using 1-way ANOVA, with repeated measures. When criteria for parametric statistics were not satisfied (i.e., normality and equal variance), a nonparametric alternative, Friedman Repeated Measures ANOVA on ranks, was used. Tukey tests were employed to further interrogate identified main effects. Relations between THC pharmacokinetic parameters and body size and composition values were explored using Pearson correlations. The level of statistical significance was set at *p* < 0.05.

## Figures and Tables

**Table 1:** Selected physiological characteristics of study participants.

Characteristic	Mean ± SD	Range
Sex (M/F)	4/3	-
Age (years)	31 ± 5	24–39
Height (cm)	170 ± 11	159–193
Body Mass (kg)	82.3 ± 17.7	62.7–113.9
Body Mass Index (kg/m^2^)	28.6 ± 6.5	23.0–40.8
Fat Mass (kg)	28.1 ± 13.5	16.3–55.6
Body Fat (%)	33.4 ± 10.1	21.2–48.9
Lean Mass (kg)	52.2 ± 10.0	38.1–66.0
Bone Mineral Content (kg)	2.3 ± 0.4	2.0–3.1

**Table 2:** Features of the commercially available edible marijuana products.

Product and Manufacturer	Nutrition	Ingredients
Ripple Blood Orange Gummies (Stillwater Brands, Commerce City, CO, USA)	20 kcal per serving: 2 gummies; (Fat 0 g, Total carbohydrate 4 g, Protein 0 g)	Glucose syrup, sugar, water, fruit juice concentrates (Apple, Pear), gelatin, modified food starch, Ripple (water, modified food starch, cannabinoid extracts, MCT oil), contains 2% or less of: natural flavors, malic acid, citric acid, carnauba wax, vegetable juice for color.
Ripple Pure 10 (Stillwater Brands, Commerce City, CO, USA)	0 kcal per serving (Fat 0 g, Total carbohydrate 0 g, Protein 0 g)	Sorbitol, modified food starch, cannabinoid extracts, MCT oil
Ripple Quick Sticks Blueberry Pomegranate (Stillwater Brands, Commerce City, CO, USA)	5 kcal per individual serving (Fat 0 g, Total carbohydrate 1 g, Protein 0 g)	Ripple (Sorbitol, Modified Food Starch, Cannabinoid Extracts, MCT Oil), Sugar, Fructose, Natural Flavors, Citric Acid, Malic Acid
Wana Fast Acting Gummies, Pina Colada Indica (Wana Brands, Boulder, CO, USA)	30 kcal per serving: 2 gummies (Fat 0 g, Total carbohydrate 8 g, Protein 0 g)	Organic Cane Sugar, Organic Tapioca Syrup, Pectin (Pectin, Potassium Sodium Tartrate, Polyphosphate, Sucrose), Citric Acid, Natural Flavoring, Sodium Citrate, Modified Food Starch, Xanthan Gum, THC.
Wana Sour Gummies (Wana Brands, Boulder, CO, USA)	15 kcal per serving (Fat 0 g, Total carbohydrate 4 g, Protein 0 g)	Organic Sugar, Organic Tapioca Syrup, Pectin (Pectin, Potassium Sodium Tartrate, Polyphosphate, Sucrose), Citric Acid, Natural Flavoring and Coloring, Sodium Citrate, Marijuana Concentrate, and Botanical Terpenes for Flavor.
Welch’s Fruit Snacks (Park Ridge, NJ, USA) *	15 kcal per serving (Fat 0 g, Carbohydrate 2 g, Sugar 3 g, Protein 0 g)	Fruit puree (grape, peach, orange, strawberry, and raspberry), corn syrup, sugar, modified corn starch, gelatin, concord grape juice from concentrate, citric acid, lactic acid, natural and artificial flavors, ascorbic acid (vitamin C), alpha tocopherol acetate (vitamin E), vitamin A palmitate, sodium citrate, coconut oil, carnauba wax, annatto (color), turmeric (color), red 40, and blue 1.

All self-administered doses of edible marijuana products contained 10 mg THC. * Marijuana-free control product. MCT: Medium chain triglycerides.

**Table 3:** Pharmacokinetic parameters for THC.

Product		T_max_ (min)	C_max_ (ng/mL)	AUC_0-240_ (min * ng/mL)	V_d_ (mL)	CL/F_0-240_ (mL/min)	k_e_ (1/min)	t_1/2_ (min)
Ripple Gummies	n	7	7	7	7	7	7	7
	$\bar{x}$	35.7	5.54	533	4,534,900	19,313	0.005	268.3
	σ	12.1	3.10	286	4,406,250	20,443	0.003	267.0
	$\bar{\mu }$	45.0	5.22	463	2,979,599	14,131	0.00	152.7
Ripple Pure 10	n	7	7	7	6	6	6	6
	$\bar{x}$	40.7	4.31	447	4,397,542	23,966	0.005	152.4
	σ	11.3	3.01	301	2,282,851	17,028	0.002	47.1
	$\bar{\mu }$	45.0	2.37	271	4,531,448	22,193	0.000	148.6
Ripple Quick Sticks	n	7	7	7	5	5	5	5
	$\bar{x}$	90.7	4.56	570	2,648,627	11,844	0.006	215.5
	σ	84.6	1.80	268	1,272,398	5691	0.004	175.0
	$\bar{\mu }$	60.0	5.17	632	2,369,956	12,915	0.000	206.3
Wana Fast Acting Gummies	n	7	7	7	6	6	6	6
	$\bar{x}$	51.4	4.39	455	4,989,024	19,431	0.005	158.8
	σ	31.1	2.91	248	4,898,405	14,590	0.002	75.5
	$\bar{\mu }$	45.0	4.29	421	2,401,279	14,491	0.010	133.5
Wana Sour Gummies	n	7	7	7	6	6	6	6
	$\bar{x}$	62.1	3.22	406	3,960,415	16,420	0.004	180.0
	σ	53.0	2.04	296	1,463,937	7526	0.001	68.3
	$\bar{\mu }$	45.0	2.57	305	4,462,147	15,837	0.000	165.5
	*P*-	0.548	0.110	0.210	0.468	0.446	0.697	0.684
	*F-*	3.061 *	2.12	1.587	0.928	0.973	0.556	0.576

T_max_: the time to maximum concentration. C_max_: the maximum concentration. AUC_0-240_: the area under the curve representing total THC exposure between time 0 and end of data collection. V_d_: the volume of distribution, an estimate of the degree to which THC is distributed in the body tissue vs. the plasma. CL/F_0-240_: the apparent total clearance of the THC from plasma after oral administration. K_e_: the rate at which the THC is removed from the body. t½: the amount of time it takes to decrease the circulating concentration to half of its initial value. All product servings contained 10 mg of THC. $\bar{x}$ represents mean value. σ represents the standard deviation. $\bar{\mu }$ represents median value. *P*- represents statistical *p*-value. *F-* represents the *F*-value from the ANOVA table unless depicted by *, in which case this is the Chi-square value.

**Table 4:** Pharmacokinetic parameters for THC-OH.

Product		T_max_ (min)	C_max_ (ng/mL)	AUC_0-240_ (min * ng/mL)	V_d_ (mL)	CL/F_0-240_ (mL/min)	k_e_ (1/min)	t_1/2_ (min)
Ripple Gummies	n	7	7	7	7	7	7	7
	$\bar{x}$	55.7	6.60	816	2,993,411	7138	0.005	512.8
	σ	16.7	3.42	361	3,760,404	2809	0.002	989.6
	$\bar{\mu }$	45.0	7.97	950	1,284,652	7100	0.000	149.3
Ripple Pure 10	n	7	7	7	7	7	7	7
	$\bar{x}$	53.6	5.05	560	3,628,286	17,124	0.005	159.4
	σ	17.0	4.20	359	2,117,850	13,390	0.001	49.0
	$\bar{\mu }$	45.0	3.32	447	3,804,011	11,432	0.000	145.8
Ripple Quick Sticks	n	7	7	7	5	5	5	5
	$\bar{x}$	100.7	5.33	700	3,036,162	8389	0.003	403.7
	σ	77.3	2.71	381	1,634,370	6850	0.003	335.4
	$\bar{\mu }$	60.0	4.62	747	3,076,976	8413	0.000	267.9
Wana Fast Acting Gummies	n	7	7	7	5	5	5	5
	$\bar{x}$	83.6	5.40	669	1,773,924	6703	0.005	213.1
	σ	49.6	3.71	361	783,269	2218	0.002	154.4
	$\bar{\mu }$	60.0	4.83	753	1,633,620	6014	0.000	138.8
Wana Sour Gummies	n	7	7	7	6	6	6	6
	$\bar{x}$	72.9	4.45	626	2,141,277	8860	0.005	234.0
	σ	48.6	2.25	310	956,697	5405	0.005	199.8
	$\bar{\mu }$	60.0	4.36	544	1,811,579	7708	0.000	189.1
	*P*-	0.369	0.390	0.065	0.758	0.169	0.975	0.778
	*F-*	4.283 *	1.076	2.553	0.468	1.808	0.117	0.440

T_max_: the time to maximum concentration. C_max_: the maximum concentration. AUC_0-240_: the area under the curve representing total THC-OH exposure between time 0 and end of data collection. V_d_: the volume of distribution, an estimate of the degree to which THC-OH is distributed in the body tissue vs. the plasma. CL/F_0-240_: the apparent total clearance of the THC-OH from plasma after oral administration. K_e_: the rate at which the THC-OH is removed from the body. t½: the amount of time it takes to decrease the circulating concentration to half of its initial value. All product servings contained 10 mg of THC. $\bar{x}$ represents mean value. σ represents the standard deviation. $\bar{\mu }$ represents median value. *P*- represents statistical *p*-value. *F-* represents the *F*-value from the ANOVA table unless depicted by *, in which case this is the Chi-square value.

**Table 5:** Pharmacokinetic parameters for THC-COOH.

Product		T_max_ (min)	C_max_ (ng/mL)	AUC_0-240_ (min * ng/mL)	V_d_ (mL)	CL/F_0-240_ (mL/min)	k_e_ (1/min)	t_1/2_ (min)
Ripple Gummies	n	7	7	7	4	4	4	4
	$\bar{x}$	105.0	44.01	7047	266,200	484	0.002	365.3
	σ	75.0	21.32	3264	140,158	147	0.000	97.3
	$\bar{\mu }$	60.0	34.28	6251	241,269	514	0.000	325.7
Ripple Pure 10	n	7	7	7	6	6	6	6
	$\bar{x}$	87.9	40.24	6311	360,519	569	0.001	846.3
	σ	67.9	19.44	3137	194,482	420	0.001	986.7
	$\bar{\mu }$	60.0	34.15	5154	299,004	568	0.000	487.8
Ripple Quick Sticks	n	7	7	7	3	3	3	3
	$\bar{x}$	130.7	42.25	6195	288,446	802	0.003	272.2
	σ	86.0	22.51	3667	28,675	344	0.001	80.7
	$\bar{\mu }$	75.0	35.63	5870	301,043	674	0.000	309.4
Wana Fast Acting Gummies	n	7	7	7	3	3	3	3
	$\bar{x}$	145.7	39.36	6467	312,251	363	0.001	611.4
	σ	84.3	15.12	2798	201,295	247	0.000	73.8
	$\bar{\mu }$	180.0	41.04	6203	208,977	262	0.000	585.7
Wana Sour Gummies	n	7	7	7	2	2	2	2
	$\bar{x}$	145.7	35.78	6009	297,051	758	0.003	677.6
	σ	68.6	18.89	3324	81,970	789	0.003	780.3
	$\bar{\mu }$	180.0	29.09	5119	297,051	758	0.000	677.6
	*P*-	0.514	0.746	0.642	0.134	0.680	0.107	0.860
	*F-*	3.270 *	1.943 *	2.514 *	2.533	0.591	2.852	0.314

T_max_: the time to maximum concentration. C_max_: the maximum concentration. AUC_0-240_: the area under the curve representing total THC-COOH exposure between time 0 and end of data collection. V_d_: the volume of distribution, an estimate of the degree to which THC-COOH is distributed in the body tissue vs. the plasma. CL/F_0-240_: the apparent total clearance of the THC-COOH from plasma after oral administration. K_e_: the rate at which the THC-COOH is removed from the body. t½: the amount of time it takes to decrease the circulating concentration to half of its initial value. All product servings contained 10 mg of THC. $\bar{x}$ represents mean value. σ represents the standard deviation. $\bar{\mu }$ represents median value. *P*- represents statistical *p*-value. *F-* represents the *F*-value from the ANOVA table unless depicted by *, in which case this is the Chi-square value.

**Table 6:** Metabolite-to-parent ratios for C_max_ and AUC_0-240_.

		THC-OH/THC		THC-COOH/THC	
Product		C_max_	AUC_0-240_	C_max_	AUC_0-240_
Ripple Gummies	n	7	7	7	7
	$\bar{x}$	1.22	1.64	10.54	17.10
	σ	0.26	0.45	7.14	12.49
	$\bar{\mu }$	1.26	1.51	6.53	11.12
Ripple Pure 10	n	7	7	7	7
	$\bar{x}$	1.11	1.35	11.46	16.06
	σ	0.24	0.53	5.31	5.53
	$\bar{\mu }$	1.02	1.21	10.52	14.55
Ripple Quick Sticks	n	7	7	7	7
	$\bar{x}$	1.28	2.06	10.84	11.76
	σ	0.58	2.35	6.63	4.58
	$\bar{\mu }$	1.21	1.33	8.83	10.81
Wana Fast Acting Gummies	n	7	7	7	7
	$\bar{x}$	1.27	1.53	13.24	17.02
	σ	0.29	0.44	7.99	7.24
	$\bar{\mu }$	1.17	1.57	11.52	18.05
Wana Sour Gummies	n	7	7	7	7
	$\bar{x}$	1.44	1.68	12.64	16.43
	σ	0.25	0.51	5.19	4.32
	$\bar{\mu }$	1.46	1.82	11.37	16.77
	*P*-	0.344	0.195	0.772	0.137
	*F-*	1.182	6.057 *	0.449	6.971 *

C_max_: the maximum concentration. AUC_0-240_: the area under the curve representing total THC-COOH exposure between time 0 and end of data collection. $\bar{x}$ represents mean value. represents the standard deviation. $\bar{\mu }$ represents median value. *P*- represents statistical *p*-value. *F*- represents the *F*-value from the ANOVA table unless depicted by *, in which case this is the Chi-square value.

**Table 7:** Pearson correlations between parameters of body composition and THC pharmacokinetics.

			Age	Ht	BMC	Fat Mass	Lean Mass	Total Mass	% Fat	BMI
T_max_	Ripple Gummies	r	0.61	0.54	0.65	0.22	0.90	0.71	−0.24	0.42
		p	0.15	0.21	0.12	0.63	0.01	0.07	0.61	0.35
	Ripple Pure 10	r	−0.16	0.91	0.87	−0.63	0.66	−0.08	−0.90	−0.54
		p	0.74	0.01	0.01	0.13	0.11	0.87	0.01	0.21
	Ripple Quick Sticks	r	−0.62	−0.20	−0.33	0.07	−0.33	−0.12	0.21	0.00
		p	0.13	0.67	0.48	0.89	0.47	0.79	0.65	1.00
	Wana Fast Acting Gummies	r	0.50	0.01	0.03	0.09	0.33	0.13	−0.14	0.10
		p	0.25	0.98	0.95	0.85	0.47	0.79	0.76	0.83
	Wana Sour Gummies	r	−0.32	0.87	0.87	−0.35	0.58	0.09	−0.53	−0.36
		p	0.47	0.01	0.01	0.45	0.17	0.85	0.22	0.43
C_max_	Ripple Gummies	r	−0.24	−0.68	−0.77	0.30	−0.80	−0.25	0.58	0.10
		p	0.61	0.09	0.04	0.51	0.03	0.58	0.17	0.82
	Ripple Pure 10	r	−0.21	−0.48	−0.60	0.35	−0.62	−0.12	0.58	0.13
		p	0.65	0.28	0.15	0.44	0.14	0.80	0.17	0.79
	Ripple Quick Sticks	r	−0.21	0.03	−0.04	−0.19	−0.31	−0.34	−0.10	−0.36
		p	0.65	0.95	0.93	0.68	0.50	0.45	0.84	0.43
	Wana Fast Acting Gummies	r	−0.07	−0.36	−0.46	0.29	−0.38	−0.00	0.31	0.18
		p	0.89	0.43	0.30	0.53	0.40	0.99	0.51	0.69
	Wana Sour Gummies	r	0.21	−0.63	−0.66	0.73	−0.42	0.29	0.84	0.60
		p	0.65	0.13	0.11	0.06	0.34	0.53	0.02	0.16
AUC_0-240_	Ripple Gummies	r	0.05	−0.63	−0.75	0.65	−0.49	0.20	0.74	0.52
		p	0.92	0.13	0.05	0.12	0.27	0.67	0.06	0.23
	Ripple Pure 10	r	−0.12	−0.34	−0.54	0.56	−0.32	0.23	0.59	0.40
		p	0.80	0.46	0.21	0.19	0.49	0.62	0.17	0.38
	Ripple Quick Sticks	r	−0.17	−0.06	−0.23	0.03	−0.25	−0.13	0.04	−0.10
		p	0.71	0.89	0.62	0.95	0.58	0.78	0.93	0.83
	Wana Fast Acting Gummies	r	0.07	−0.43	−0.52	0.39	−0.34	0.10	0.38	0.32
		p	0.88	0.34	0.24	0.39	0.46	0.83	0.40	0.49
	Wana Sour Gummies	r	0.35	−0.47	−0.52	0.89	−0.13	0.59	0.83	0.82
		p	0.44	0.29	0.23	0.01	0.79	0.16	0.02	0.02

*n* = 7 for all cells. Ht: Height. BMC: Bone Mineral Content. BMI: Body Mass Index. AUC_0-240_: Area under the concentration curve between time 0 and 240 min (4-h).

**Table 8:** LC-MS/MS ion transitions monitored for danysl derivatives of cannabinoids in human plasma.

Analyte Name	Precursor Ion	Product Ion	Frag (V)	CE (V)	Cell Acc (V)	Polarity
Dansyl-THC-COOH	343.2	532	156	20	4	Positive
Dansyl-THC-COOH	343.2	227	156	56	4	Positive
Dansyl-THC-COOH-D9	587.3	233	156	56	4	Positive
Dansyl-THC-COOH-D9	587.3	170	156	40	4	Positive
Dansyl-THC-OH	564.3	241	139	64	4	Positive
Dansyl-THC-OH	564.3	256	139	36	4	Positive
Dansyl-THC-OH-D3	567.3	241	139	64	4	Positive
Dansyl-THC-OH-D3	567.3	256	139	36	4	Positive
Dansyl-THC	548.3	171	160	44	4	Positive
Dansyl-THC	548.3	156	160	80	4	Positive
Dansyl-THC-D3	551.3	171	160	44	4	Positive
Dansyl-THC-D3	551.3	156	160	80	4	Positive

## Data Availability

The data presented in this study are available on request from the corresponding author.
